# MMP‐1 and MMP‐9 regulate epidermal growth factor‐dependent collagen loss in human carotid plaque smooth muscle cells

**DOI:** 10.1002/phy2.224

**Published:** 2014-02-10

**Authors:** Velidi H. Rao, Vikash Kansal, Samantha Stoupa, Devendra K. Agrawal

**Affiliations:** 1 Department of Biomedical Sciences, Creighton University School of Medicine, Omaha, 68178, Nebraska; 2 Center for Clinical and Translational Science, Creighton University School of Medicine, Omaha, 68178, Nebraska

**Keywords:** Atherosclerosis, carotid plaques, interstitial collagens, matrix metalloproteinase, vascular smooth muscle cells

## Abstract

Mechanisms underlying the rupture of atherosclerotic plaque, a crucial factor in the development of myocardial infarction and stroke, are not well defined. Here, we examined the role of epidermal growth factor (EGF)‐mediated matrix metalloproteinases (MMP) on the stability of interstitial collagens in vascular smooth muscle cells (VSMCs) isolated from carotid endarterectomy tissues of symptomatic and asymptomatic patients with carotid stenosis. VSMCs isolated from the carotid plaques of both asymptomatic and symptomatic patients were treated with EGF. The MMP‐9 activity was quantified by gelatin zymography and the analysis of mRNA transcripts and protein for MMP‐9, MMP‐1, EGFR and collagen types I, Col I(*α*1) and collagen type III, Col III(*α*1) were analyzed by qPCR and immunofluorescence, respectively. The effect of EGF treatment to increase MMP‐9 activity and mRNA transcripts for MMP‐9, MMP‐1, and EGFR and to decrease mRNA transcripts for Col I(*α*1) and Col III(*α*1) was threefold to fourfold greater in VSMCs isolated from the carotid plaques of symptomatic than asymptomatic patients. Inhibitors of EGFR (AG1478) and a small molecule inhibitor of MMP‐9 decreased the MMP9 expression and upregulated Col I(*α*1) and Col III(*α*1) in EGF‐treated VSMCs of both groups. Additionally, the magnitude in decreased MMP‐9 mRNA and increased Col I(*α*1) and Col III(*α*1) due to knockdown of MMP‐9 gene with siRNA in EGF‐treated VSMCs was significantly greater in the symptomatic group than the asymptomatic group. Thus, a selective blockade of both EGFR and MMP‐9 may be a novel strategy and a promising target for stabilizing vulnerable plaques in patients with carotid stenosis.

## Abstract (precis)

This report described the underlying mechanisms by which MMP‐1 and MMP‐9 induced by EFGR activation decreases the interstitial collagens and this could result in plaque instability in patients with carotid stenosis. Thus, selective blockade of EGFR and/or MMP‐9 may be a novel strategy and a promising target to stabilize atherosclerotic plaques and thus decreases morbidity and mortality.

## Introduction

Atherogenesis is a chronic, progressive process that develops over several decades (Peeters et al. 2009; Leeuwis et al. 2010). Fatalities associated with thrombotic complications in atherosclerosis are increasing worldwide. Atherosclerotic plaques with a thin fibrous cap are prone to rupture that may lead to thromboembolism and subsequent ischemic stroke, whereas plaques with a thick cap are more stable and have a lower complication rate (Dhume and Agrawal 2003). The atherosclerotic plaques are largely composed of vascular smooth muscle cells (VSMCs), macrophages and T lymphocytes, and are characterized by the deposition of lipids, cholesterol, calcium, and cellular debris within the intima of the vessel wall (Vukovic et al. 2006). Changes in the composition of the extracellular matrix (ECM) play an important role in the atherogenic process. The balance between the matrix accumulation and degradation in the ECM determines the outcome of plaque stability (Adiguzel et al. 2009; Murillo et al. 2009; Newby et al. 2009).

Reorganization of the ECM is a key characteristic of hypertensive vascular remodeling. ECM macromolecules, particularly fibrillar interstitial Col I(*α*1) and Col III(*α*1) mainly synthesized by VSMCs, are the major components of atherosclerotic plaques and provide tensile strength to the fibrous cap in the plaque (Adiguzel et al. 2009; Murillo et al. 2009; Newby et al. 2009). Col I(*α*1) provides the structural support for all tissues and organs, including atherosclerotic plaque (Luan et al. 2003; Newby 2006; Adiguzel et al. 2009; Newby et al. 2009). Collagenases of the MMP family (MMP‐1, MMP‐8, and MMP‐13) initiate the first steps in the degradation of native Col I(*α*1), and Col III(*α*1), resulting in the generation of three‐quarter and one‐quarter length fragments (Herman et al. 2001). The resulting fragments are further degraded by gelatinases and MMP‐3 leading to less resistance to mechanical stresses (Luan et al. 2003). MMP‐9 is upregulated in VSMCs present in atheroma (Murillo et al. 2009; Peeters et al. 2009). In restenotic lesions, various cytokines and growth factors EGF, PDGF, TGF, VEGF, and angiotensin II (Luan et al. 2003; Newby 2006; Louis and Zahradka 2010) secreted by atherosclerotic carotid plaque are involved in cellular events such as proliferation, migration, and apoptosis and may regulate the activity and expression of MMPs and tissue inhibitor of metalloproteinases (TIMPs). The EGFR is a receptor protein tyrosine kinase (PTK) and is involved in various cellular processes and diseases, but its role in atherosclerosis is less understood. A number of studies have revealed that EGFR and its family of ligands, including EGF, are present in human carcinomas, but only a few studies have identified their presence in atherosclerotic plaques (Dreux et al. 2006; Normanno et al. 2006; Stanic et al. 2012). In the present study, we investigated the functional role of EGF and MMPs on interstitial collagens in relation to plaque stability in human carotid plaques as well as in VSMCs isolated from AS and S patients with carotid stenosis.

## Methods

### Carotid endarterectomy specimens and culture of VSMCs

Carotid plaques from patients undergoing carotid endarterectomy were collected. These patients were deemed appropriate based on American Heart Association (AHA) criteria that define the risk–benefit ratio in AS and S carotid disease. The Institutional Review Board of Creighton University approved the exempted research protocol because the carotid endarterectomy specimens were truly anonymized. No specimen was marked or identified by social security number, medical record number, pathology accession number, or any other similar number or code. Thus, the specimens could not be linked to living individuals or with associated medical information. No personnel involved in this study could identify the subjects from whom the specimens were obtained.

The carotid plaques were categorized by the surgeon as either AS or S from the history and clinical evaluation of patients. Symptoms included hemispheric transient ischemic attacks, amaurosis fugax, or stroke (Dhume and Agrawal 2003; Vukovic et al. 2006). The categorization of carotid stenosis as “symptomatic/unstable” or “asymptomatic/stable” was provided to laboratory investigators in an anonymous manner. The specimens were collected fresh in the University of Wisconsin solution and transported within 2–3 h to the laboratory, where all procedures were carried out under sterile conditions.

VSMCs were prepared from carotid plaques by an established method developed in our laboratory (Dhume and Agrawal 2003). After gently scraping endothelial and adventitial layers, the medial layer was homogenized, washed in serum‐free DMEM (Gibco BRL, Grand island, NY) and digested with 0.025% trypsin for 30 min at 37°C followed by 0.1% collagenase (Sigma, St. Louis, MO) digestion for 3 h. The pellet was suspended in smooth muscle cell medium (ScienCell, Carlsbad, CA) and seeded on to 25 cm^2^ culture flasks and maintained at 37°C and 5% CO_2_. The cells from the second through fifth passages were used. The phenotype and the homogeneity of isolated smooth muscle cells (SMCs) were confirmed by positive staining for smooth muscle *α*‐actin and caldesmon, as reported previously (Jia et al. 2010).

### Tissue extraction, cell culture, and treatment protocol

Tissue samples were extracted in 50 mmol/L Tris, 0.2% Triton X‐100, 10 mmol/L CaCl_2_ pH 7.5 as previously described (Rao et al. 2003, 2012; Dreux et al. 2006). Volumes of the tissue extracts were adjusted to obtain equal protein content and analyzed by gelatin zymography. VSMCs at preconfluency were incubated in serum‐free medium containing EGF at10 ng/mL for 24 h. To confirm the activation of EGFR, cells were pretreated at 10 *μ*mol/L and 15 *μ*mol/L AG1478, an inhibitor of EGFR (AG Scientific, San Diego, CA) for 30 min and then treated for 24 h with EGF (10 ng/mL) in serum‐free medium. Cells from carotid plaques were also pretreated with a selective MMP‐9 inhibitor (Calbiochem, Billerica, MA) at 5, 25, and 50 nmol/L for 30 min before the incubation in the presence or absence of EGF (10 ng/mL) for 24 h.

### Gelatin zymography

Tissue samples were extracted in 50 mmol/L Tris, 0.2% Triton X‐100, 10 mmol/L CaCl_2_ pH 7.5 as described earlier (Dreux et al. 2006; Rao et al. 2012). VSMCs (2.5 × 10^6^ cells/well) were seeded in a 6‐well tissue culture plate and incubated in 1.0 mL of serum‐free medium for 24 h. The medium was collected, centrifuged to remove debris, and stored at −20°C. An equal volume of conditioned medium from SMCs grown in serum‐free medium was used for gelatin zymography (Rao et al. 2003). Briefly, samples were run on 8% sodium dodecyl sulfate polyacrylamide gel electrophoresis (SDS‐PAGE) containing gelatin (1.0 mg/mL). After electrophoresis, the gels were washed in Triton X‐100 and incubated for 18 h in 50 mmol/L Tris‐HCl buffer (pH 7.5) containing 0.2 mol/L NaCl and 10 mmol/L CaCl_2_. Gels were stained with Brilliant Blue R250 and destained. Gelatinolytic activity of MMP‐9 was evident as a clear band against the blue background of the stained gel.

To confirm whether or not the clarified zones on the gelatin zymogram were due to MMP‐9 or serine proteases, duplicate gels were run and incubated in proteolysis buffer with the addition of appropriate inhibitors for the enzyme class. The inhibitors included 10 mmol/L ethylenediaminetetraacetic acid (EDTA) for MMPs, and 1.0 mmol/L phenylmethylsulfonyl fluoride (PMSF) for serine proteases (Rao et al. 2012).

### Immunofluorescence staining

Cryosections (5 *μ*m) from both S and AS carotid plaques were air‐dried onto microscope slides, fixed by immersion in ice‐cold acetone for 5 min and subjected to immunofluorescence microscopy, as described previously (Rao et al. 2012), using rabbit polyclonal antibodies for MMP‐9 (kindly provided by Dr. Z. Smith, University of Florida) at 1:200 dilution. Antibodies to MMP‐1 (rabbit polyclonal), EGFR (rabbit polyclonal) and Col I(*α*1) (rabbit polyclonal) were purchased from Santa Cruz Biotechnology and used at 1:100 dilution. In brief, the frozen sections were kept in phosphate buffered saline (PBS) for 5 min followed by incubation at room temperature in blocking solution containing serum and triton X‐100 for 1 h. Primary antibodies were allowed to bind overnight at 4°C. After washing with PBS, the tissue sections were incubated with Alexa595‐conjugated secondary antibody (Invitrogen, Grand Island, NY) or cy3 dye at 1:200 for 2 h at room temperature. The slides were washed with PBS, stained with 4,6‐diamidino‐2‐phenylindole (DAPI) before placing the coverslip. The immunofluorescence was observed in an Olympus inverted fluorescent microscope (Olympus BX51; Olympus America, Center Valley, PA). Negative controls were run by omitting the primary antibody (data not shown). The immunofluorescence staining for Col I(*α*1) was confirmed in frozen sections from AS and S carotid plaques by using Masson's trichrome staining (Richard‐Allan Scientific, Campus Drive Kalamazoo, MI).

### RNA isolation, cDNA synthesis, and real‐time PCR

Total RNA was isolated using Trizol reagent (Sigma) from tissues and VSMCs according to the manufacturer's instructions. The yield of RNA was quantified using a Nanodrop (Thermo‐Scientific, Rockford, IL). First‐strand cDNA synthesis was done following the manufacturer's instructions (Improm II reverse transcription kit; Promega, Madison, WI) using oligo dT primers. Real‐time PCR was done using SYBR Green Master Mix and a Real‐time PCR system (CFX96; BioRad Laboratories, Hercules, CA). The primers for different genes were obtained from Integrated DNA Technologies (Coralville, IA). The specificity of the primers was checked by running a melting curve. The PCR cycling conditions included 5 min at 95°C for initial denaturation, 40 cycles of 30 sec at 95°C, 30 sec at 55–60°C (depending upon the primer annealing temperatures), and 30 sec at 72°C. Fold expression of mRNA transcripts relative to controls was determined after normalizing to GAPDH. A complete list of the primers and their sequences is provided in Table 1.

**Table 1.** Primer sequences used for qRT‐PCR

Primer name	Sequence
GAPDH (Forward)	5′‐GAA ACC TGC CAA GTA TGA TGA C‐3′
GAPDH (Reverse)	5′‐ACC TGG TCC TCA GTG TAG C‐3′
MMP‐1 (Forward)	5′‐TGC AAC TCT GAC GTT GAT CCC AGA‐3′
MMP‐1 (Reverse)	5′‐ACT GCACAT GTG TTC TTG AGC TGC‐3′
MMP‐9 (Forward)	5′‐ATT TCT GCC AGG ACC GCT TCT ACT‐3′
MMP‐9 (Reverse)	5′‐CAG TTT GTA TCC GGC AAA CTG GCT‐3′
EGFR (Forward)	5′‐AGG AAG AAG CTT GCT GGT AGC ACT‐3′
EGFR (Reverse)	5′‐TTT GCA GTG GAA GCC TTG AAG CAG‐3′
Col I(*α*)1 (Forward)	5′‐CAA TGC TGC CCT TTC TGC TCC TTT‐3′
Col I(*α*)1 (Reverse)	5′‐CAC TTG GGT GTT TGA GCA TGG CCT‐3′
Col I(*α*)2 (Forward)	5′‐GGC AAA CAT GGA AAC CGT GGT GAA‐3′
Col I(*α*)2 (Reverse)	5′‐GGC AGA CCT TGC AAT CCA TTG TGT‐3′
Col III(*α*)1 (Forward)	5′‐TAT CGA ACA CGC AAG GCT GTG AGA‐3′
Col III(*α*)1 (Reverse)	5′‐GGC CAA CGT CCA CAC CAA ATT CTT‐3′

### Cell transfection

VSMCs isolated from both AS and S plaques were plated in six‐well plates and grown to 60–80% confluency in smooth muscle cell medium without antibiotics. The cells were transfected with either 40 nmol/L MMP‐9 siRNA or scrambled control siRNA (Santa Cruz Biotechnology, Santa Cruz, CA) using Lipofectamine 2000 (Invitrogen) adhering to the manufacturer's instructions for 6 h. The cells were allowed to recover for 24 h in the medium supplemented with 20% bovine serum albumin. The cells were then stimulated with or without EGF (10 ng/mL) for 24 h in serum‐free medium. After harvesting the cells, qPCR was done to quantify mRNA expression of Col I(*α*1), Col III(*α*1), MMP‐1, MMP‐9, EGFR and GAPDH genes using the primers listed in Table 1.

### Statistical analysis

All data are expressed as mean ± SD from three independent experiments using carotid endarterectomy tissues from individual patients (*N* = 3 in each group). Statistical analysis was performed using Student's *t* test between the tissues or VSMCs from asymptomatic and symptomatic plaques. Multiple group comparison was performed using analysis of variance. A *P* value of <0.05 was considered significant.

## Results

### Activity and mRNA expression of MMP‐9 and MMP‐1 is increased in AS and S human carotid plaques and VSMCs

**Figure 1.**
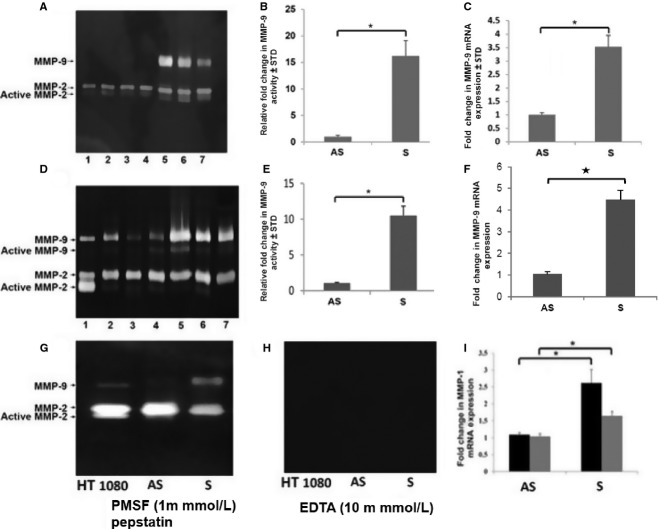
EGF modulates MMP‐9 and MMP‐1 mRNA expression. Serum‐free media from VSMCs (A, B) and tissue extracts (D, E) were analyzed for MMP‐9 activity and mRNA transcripts (C, F). The RNA isolated from cell lysates and tissue extracts were subjected to qPCR and the results were expressed as fold change compared to AS group (C, F). Lane1, HT 1080 (positive control); lanes 2–4, AS and lanes 5–7, S. The MMP activity is abolished with EDTA, an inhibitor of MMPs (H) but not with PMSF, a serine or aspartate inhibitor (G). Data are shown as mean ± SD *P < 0.01. *N* = 3.

The mRNA expression of MMP‐1 was significantly upregulated in both isolated VSMCs and tissue carotid plaques from S patients (Fig. 1I). The latent and active forms of MMP‐9 were detected by gelatin zymography in isolated VSMCs (Fig. 1A) and tissue extracts (Fig. 1D) of S and AS plaques. Densitometric analysis of gelatin zymograms showed significantly higher MMP‐9 activity in S plaque than in AS plaque. The mRNA expression of MMP‐9 and MMP‐1 was also significantly upregulated in both isolated VSMCs and tissue carotid plaques of S patients (Fig. 1C, F, and I). The activity of MMP‐9 was completely abolished by treatment with EDTA, which is an inhibitor of MMPs (Fig. 1H) but not an inhibitor of serine proteases (PMSF) (Fig. 1G).

### Increased immunostaining for MMP‐9, MMP‐1, and EGFR in tissue sections from symptomatic plaques

**Figure 2.**
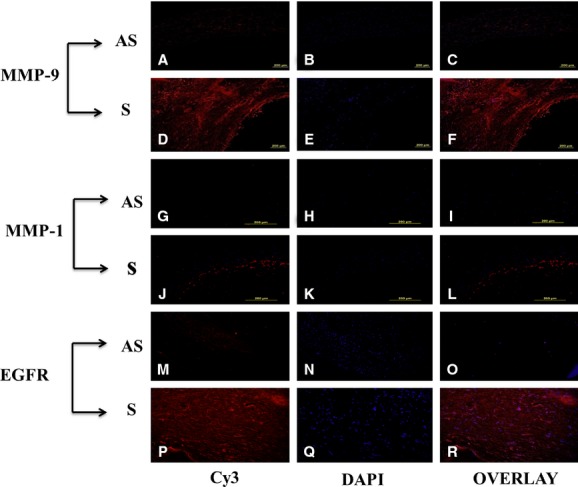
Immunofluorescence staining of MMP‐9, MMP‐1, and EGFR in carotid plaques. The immunoreactivity of MMP‐9 and MMP‐1 was greater in the symptomatic plaques as compared to AS (D, J). This is a representative of three individual tissues in each group. These results are in agreement with mRNA transcripts for MMP‐9 and MMP‐1 in tissue extracts and VSMCs isolated from human carotid plaques (C, F). Similarly, the EGFR immunostaining is also increased in symptomatic plaques (P), which confirms our results on greater expression of EGFR mRNA transcripts in S compared to AS (Fig. 3E). Data are shown as mean ± SD; *P < 0.01. *N* = 3

**Figure 3.**
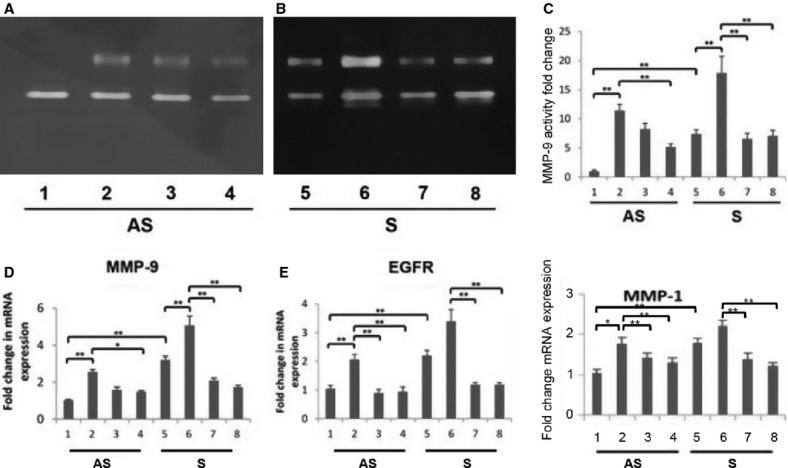
EGFR inhibitor, AG1478 modulate MMP‐9 activity and mRNA expression of MMP‐9, EGFR, and MMP‐1 in EGF‐treated VSMCs. Cultured VSMCs from AS and S were serum starved for 1 h followed by treatment with or without EGF (10 ng/mL) for 24 h in the presence or absence of AG1478. The MMP‐9 activity (A–C) and the RNA isolated from cell lysates was subjected to qPCR (D: MMP‐9; E: EGFR; F: MMP‐1) and the results were expressed as fold change compared to AS group. Lanes 1‐4, AS; lanes 5‐8, S; lanes 2 and 6, EGF treated; lanes 3 and 4 (AS) and lanes 7 and 8 (S), EGF treated in combination with AG1478 (10 and 15 *μ*mol/L). Data are shown as mean ± SD; **P < 0.05. *N* = 3

The immunoreactivity of MMP‐9 (Fig. 2D) was greater in tissue sections of S compared to AS plaques (Fig. 2A). The immunoreactivity of MMP‐1 was also more intensified in the tissue sections of S plaques (compare Fig. 2). These results are in agreement with mRNA transcripts for MMP‐9 and MMP‐1 in carotid plaques and VSMCs isolated from human carotid plaques (Fig. 1C and F). The EGFR immunostaining was also increased in S plaque sections (Fig. 2P), which confirms our results regarding a greater expression of EGFR mRNA transcripts in S compared to AS plaques (Fig. 3E). Negative controls omitting the primary antibody showed no background expression (data not shown). These immunofluorescence studies therefore confirm the results of the activity and expression of MMP‐9, MMP‐1, and EGFR in both VSMCs and carotid plaques.

### EGF upregulates MMP‐9, MMP‐1, and EGFR

VSMCs isolated from both AS and S plaques were examined for changes in proteolytic activity and mRNA expression of MMP‐9 and mRNA transcripts of MMP‐1 with and without EGF treatment (10 ng/mL) for 24 h. The gelatinolytic activity, mRNA transcripts of MMP‐9 and MMP‐1 were significantly increased (12‐, 3.5‐ and twofold, respectively) in VSMCs isolated from S carotid plaques compared to AS (Fig. 3A–D). The increase in the mRNA transcripts of EGFR and MMP‐1 in response to EGF was twofold in VSMCs from S compared to that of AS plaques (Fig. 3E–F).

### EGFR mediates regulation of MMP‐1 and MMP‐9 in Plaque VSMCs

To demonstrate the effect of EGF specifically via EGFR, an EGFR inhibitor – AG1478 – was used to examine MMP‐1 and MMP‐9 expression. VSMCs isolated from both AS and S plaques were treated with EGF (10 ng/mL) alone or in combination with AG1478 (10 and 15 *μ*mol/L) for 24 h in serum‐free medium. The increased activity and mRNA transcripts for MMP‐1 and MMP‐9 in EGF‐treated VSMCs from AS and S carotid plaques were significantly decreased with AG1478 (Fig. 3A–D and F). The expression of EGFR in EGF‐treated cells was also decreased in VSMCs treated with AG1478 in a dose‐dependent manner (Fig. 3E).

### Modulation of Col I(α1) and Col III(α1) expression in Plaque VSMCs

**Figure 4.**
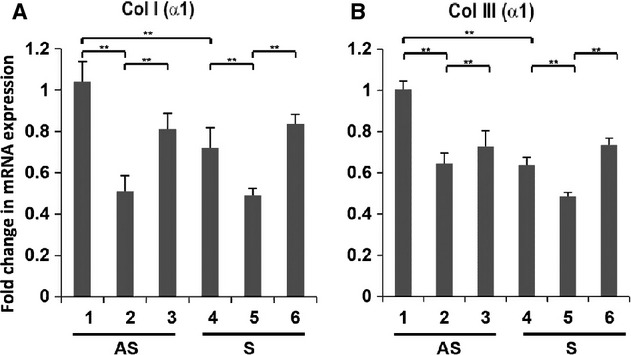
EGFR inhibitor AG1478 modulates the mRNA expression of Col I (*α*1) and Col III (*α*1) in EGF‐treated VSMCs. VSMCs were pretreated with AG1478 for 30 min followed by treatment with EGF (10 ng/mL) alone or in combination with AG1478 (15 *μ*mol/L) for 24 h in serum‐free medium. The mRNA isolated from cell lysate was subjected to qPCR and the results were expressed as fold change compared to AS group. Lanes 1‐3, AS; lanes 4‐6, S; Lanes 1 and 4, untreated; lanes 2 and 5, EGF treated; lanes 3 and 6, EGF treated in combination with AG1478 (15 *μ*mol/L). Data are shown as mean ± SD; **P < 0.05. *N* = 3

To determine the regulation of collagen types I and III by EGF, VSMCs were treated for 24 h with EGF in the presence or absence of EGFR inhibitor (15 *μ*mol/L AG1478) and the mRNA transcripts for Col I(*α*1) and Col III(*α*1) were quantified. The mRNA transcripts for collagen type I and type III were significantly lower in S compared to AS plaque VSMCs (Fig. 4A and B). The EGF treatment significantly decreased the mRNA expression of Col I(*α*1) and Col III(*α*1) in VSMCs from both S and AS plaques (Fig. 4A and B). The effect of EGF to decrease the expression of mRNA transcripts of both Col I(*α*1) and Col III(*α*1) was inhibited by AG1478 (15 *μ*mol/L). These results demonstrate that EGFR regulates the expression of type I and III collagens in plaque VSMCs.

### Effect of MMP‐9 inhibitor on the expression of collagen types I and III mRNAs in VSMCs

**Figure 5.**
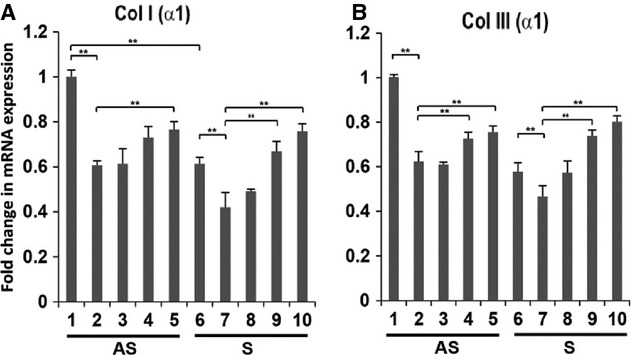
MMP‐9 Inhibitor (MI) modulates the mRNA expression of Col I (*α*1) and Col III (*α*1) in EGF‐treated VSMCs. VSMCs were pretreated with MI for 30 min followed by treatment with EGF (10 ng/mL) alone or nation with MI. The mRNA isolated from VSMCs was subjected to qPCR and the results were expressed as fold change compared to AS group. Lanes, 1‐5, AS; lanes 6‐10, S; lanes 1 and 6, untreated; 2 and 7, EGF treated (10 ng/mL); lane 3‐5 and 8‐10, EGF‐treated VSMCs in combination with MI at 5 nmol/L (lanes 3 and 8); 25 nmol/L (lanes 4 and 9); and 50 nmol/L (lanes 5 and 10). Results were expressed as fold change compared to AS group. Data are shown as mean ± SD; *P < 0.05. *N* = 3

We examined whether or not the expression of collagen type I and type III in EGF‐treated VSMCs is regulated by a small molecule inhibitor of MMP‐9. VSMCs isolated from human carotid plaques were treated for 24 h with MMP‐9 inhibitor at doses varying from 5–50 nmol/L, with and without EGF (10 ng/mL). The EGF‐induced inhibition in mRNA transcripts for Col I(*α*1) and Col III(*α*1) in plaque VSMCs of both AS and S groups was significantly protected in the presence of both 25 and 50 nmol/L of MMP‐9 inhibitor (Fig. 5A and B).

### Effect of MMP‐9 siRNA on collagen types I and III, EGFR and MMP‐9 mRNAs in VSMCs

**Figure 6.**
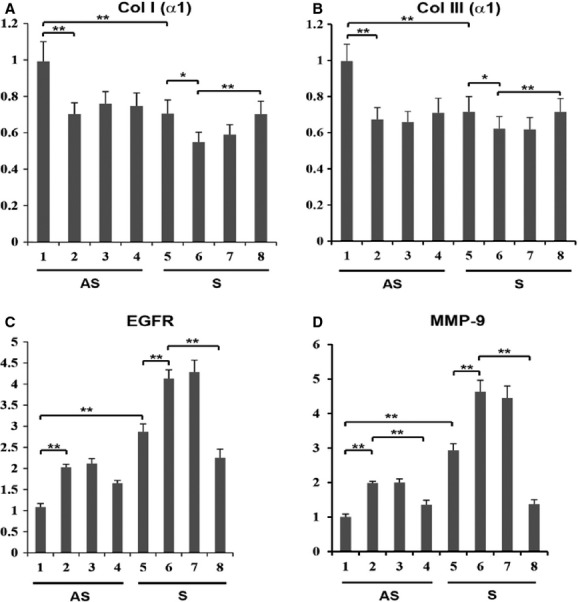
Knockdown of MMP‐9 gene on the expression of Col I (*α*1), Col III (*α*1), EGFR and MMP9 in VSMCs treated with EGF. VSMC cells isolated from carotid plaques transfected with either MMP‐9 siRNA or nonspecific control siRNA followed by treatment with EGF for 18 h. The mRNA isolated from cell lysate was subjected to qPCR and the results were expressed as fold change compared to AS group. Lanes, 1‐4, AS; lanes 5‐8, S; Lanes 1 and 4, untreated; lanes 2 and 6, EGF treated; lane 3 and 5, control siRNA (40 nmol/L) treated with EGF; and lanes 4 and 8, MMP‐9 siRNA (40 nmol/L) treated with EGF. Data are shown as mean ± SD; *P < 0.05. *N* = 3

Next, we examined whether or not MMP‐9 siRNA transfection modulates Col I(*α*1) and Col III(*α*1) expression in EGF‐treated VSMCs. The mRNA transcripts for Col I(*α*1) and Col III(*α*1) were significantly decreased in S compared to AS plaque VSMCs. The EGF treatment further decreased expression in VSMCs of both S and AS groups. Transfection of VSMCs isolated from both S and AS carotid plaque with MMP‐9 siRNA followed by treatment with EGF increased expression of collagen types I and III (Fig. 6A and B). These results are in agreement with those of MMP‐9 inhibitor in EGF‐treated VSMCs on mRNA transcripts for interstitial collagens, as shown in previous section. In addition, the mRNA transcripts for MMP‐9 and EGFR were also significantly decreased with MMP‐9 siRNA in VSMCs treated with EGF (Fig. 6C and D).

### Immunofluorescence staining of collagen I Col I (α1) is decreased in tissue sections from S compared to AS carotid plaques

**Figure 7.**
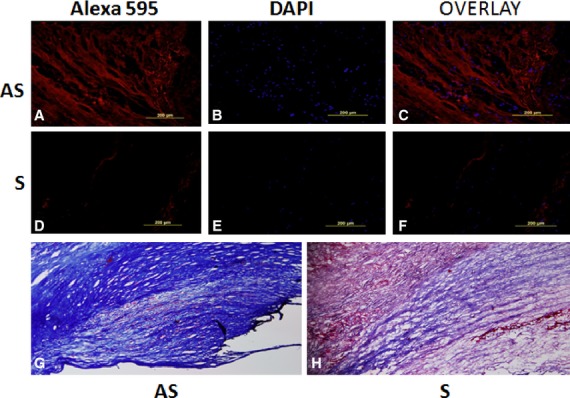
Immunofluorescence staining of collagen I, Col I (*α*1) in tissue sections of S and AS carotid plaques. The immunofluorescence staining of collagen I was greater in AS tissue sections (A, C) than in S carotid plaques (D, F). B and E: DAPI (blue) was used to stain nuclei. Negative controls with primary antibody omitted showed no background expression. Masson's Trichrome Staining in AS (G) and S (H) for collagen confirmed immunofluorescence staining.

The immunofluorescence staining of collagen I was greater in AS tissue sections (Fig. 7A) than in S carotid plaques (Fig. 7D). These results are in agreement with Col I(*α*1) mRNA transcripts in VSMCs isolated from human carotid plaques (Fig. 4A). Negative controls with the omission of primary antibody showed no background staining (data not shown). The Masson's trichrome staining for collagen also revealed much more decreased collagen in S tissue sections (Fig. 7H) than in AS plaques (Fig. 7G). These studies reveal that collagen is lower in S carotid plaques than in AS carotid plaques.

**Figure 8.**
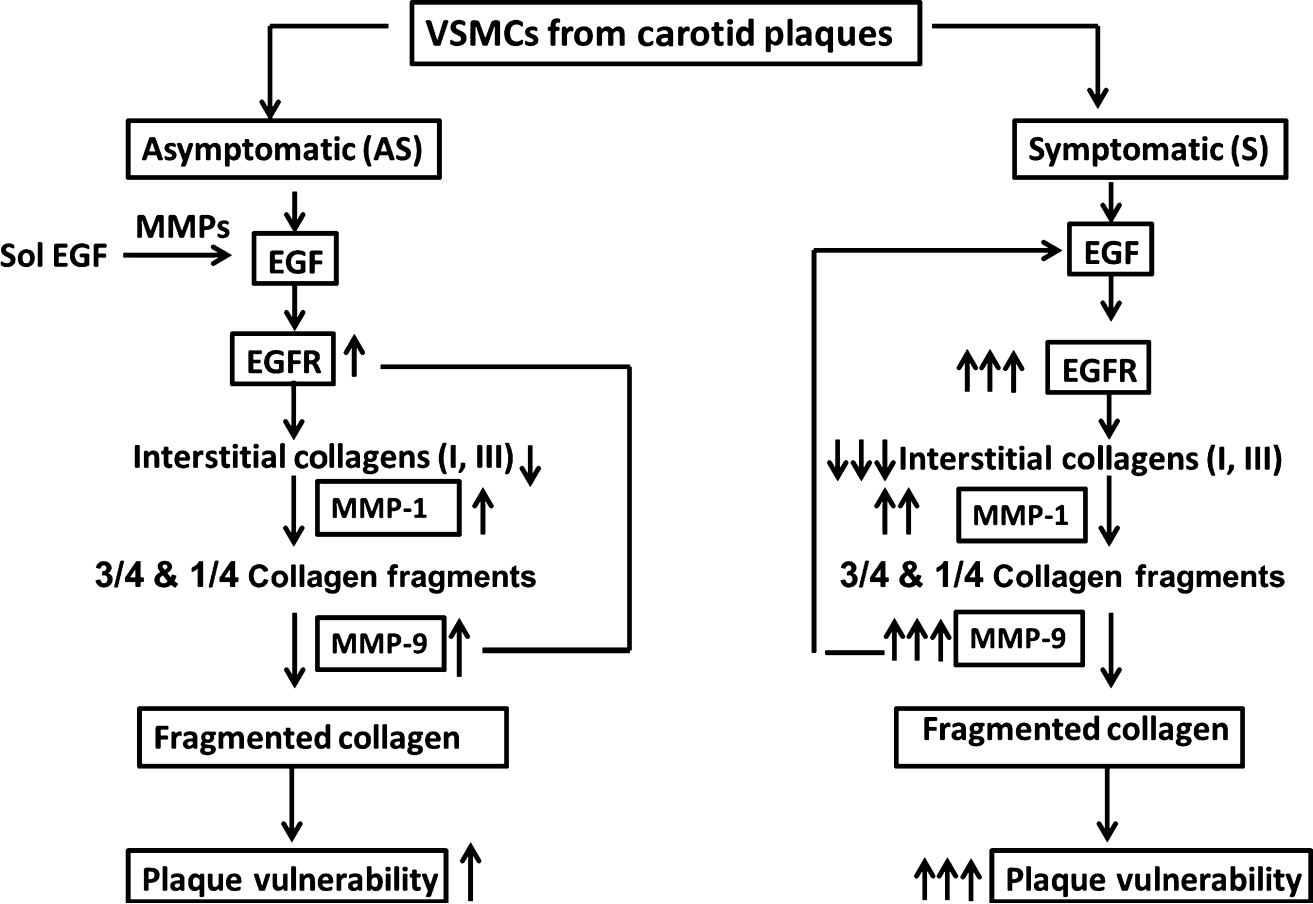
A schematic diagram showing the role of EGFR in the degradation of fibrillar collagens in involving MMPs (MMP‐1 and MMP‐9) in VSMCs isolated from S and AS carotid plaques.

## Discussion

The arterial wall provides an environment in which tissue development can maintain organ morphology and function. The main cellular components in restenotic lesions are SMCs and macrophages (Dhume and Agrawal 2003). Extracellular matrix mainly composed of Col I(*α*1), Col III(*α*1) and elastin plays an important role in the behavior of both primary and restenotic lesions in the vascular wall (Dab et al. 2009); (Dab et al. 2012); (Moreno et al. 1994; Luan et al. 2003). It is suggested that the increased matrix is associated with plaque stability while its degradation leads to the rupture of the fibrous cap (Dhume and Agrawal 2003).

Atherosclerosis is an inflammatory and progressive disease of arterial wall. The inflammatory infiltrate around the plaque rupture site is believed to be responsible for the destabilization of vulnerable plaque (Buja and Willerson 1994; Moreno et al. 1994; Libby and Aikawa 2003; Molloy et al. 2004). Ruptured plaque in human atheroma has a thin, collagen fibrous cap and a macrophage‐rich lipid core (Libby and Aikawa 2003; Adiguzel et al. 2009). Increased collagen content has been reported in collagenase‐resistant mutant knock‐in mice with atherosclerosis to suggest a critical role for collagenolysis in the turnover of collagen in plaques (Fukumoto et al. 2004). The atherosclerotic plaque remodeling and risk for plaque rupture in symptomatic plaques are partially mediated by MMPs (Loftus et al. 2002; Molloy et al. 2004; Eldrup et al. 2006; Kunte et al. 2008; Peeters et al. 2009, 2011). It is reported that thinning and weakening of the fibrous cap due to a decreased amount of Col I(*α*1), the major load‐bearing molecule, is the mechanism that renders atheroma prone to rupture (Shah and Galis 2001; Fuster et al. 2005; Schmidt et al. 2006). The cleaved fragments of interstitial collagens by collagenases become accessible to other MMPs, including MMP‐3 and MMP‐9 (Sukhova et al. 1999). These interstitial collagenases are increased in atheromatous (vulnerable) compared to fibrous (stable) plaques. Endothelial cells, SMCs, and macrophages express collagenases and colocalize with type I collagen within the shoulder region of the plaque (Herman et al. 2001; Cheng et al. 2009; Peeters et al. 2011; Quillard et al. 2011).

Here, we found that the expression of MMP‐1 is significantly increased in VSMCs isolated from S patients compared to AS patients with carotid stenosis. The activity and the expression of MMP‐9 were also significantly increased in both carotid plaque tissues and isolated VSMCs from AS patients compared to those of the S patients, thus confirming the findings of previous studies in atherosclerotic lesions (Nikkari et al. 1995; Shah and Galis 2001; Loftus et al. 2002; Molloy et al. 2004; Sluijter et al. 2006; Kunte et al. 2008; Peeters et al. 2011; Quillard et al. 2011). The MMP‐9 is also associated with atherosclerotic plaque development, inflammation, and decreased VSMC cell content (Godin et al. 2000; Laxton et al. 2009). However, this is the first investigation on the systematic comparison of AS and S carotid plaques in relation to both MMP‐1 and MMP‐9.

Several proatherosclerotic cytokines and growth factors, including tumor necrosis factor‐*α*, interleukin‐1*β*, vascular endothelial growth factor, platelet‐derived growth factor‐BB (PDGF‐BB), and basic fibroblast growth factor may regulate the activity and expression of MMPs in atherosclerotic carotid plaque (Galis et al. 1994; Ardissino et al. 1997; Bond et al. 1998; Tuomainen et al. 2007). Though EGFR regulate the expression of several MMPs in a variety of human tumors (Lynch et al. 2004; Ning et al. 2005; Cowden Dahl et al. 2007), its role in the regulation of MMP‐1, MMP‐9, and EGFR in relation to the stability of collagens in atherosclerotic plaques is not well understood. The EGF system has been shown to play an important role in regulation of normal heart physiology during development as well as in the postnatal heart (Holbro et al. 2003; Johnston et al. 2006). Both macrophages and SMCs express EGFR, but the functional significance of this receptor is not clearly known in atherosclerosis. The EGFR ligands, heparin‐binding epidermal growth factor (HB‐EGF), EGF and betacellulin, have been reported to be present in atherosclerotic plaque and may play a major role in carotid plaque vulnerability (Kagiyama et al. 2002; Holbro et al. 2003; Iwamoto and Mekada 2006; Johnston et al. 2006). In the present study, a significant increase in the expression of EGFR was observed in EGF‐treated VSMCs isolated from S plaques compared to AS plaques with carotid stenosis. This prompted us to investigate the role of EGF, the ligand for EGFR, in the regulation of MMP‐1 and MMP‐9 in VSMCs isolated from both S and AS on carotid plaque stability. This is the first report on the role of MMP‐1 and MMP‐9 in the regulation of EGF‐dependent collagen loss in human carotid plaque SMCs.

Currently available broad‐spectrum inhibitors have minimal effect on plaque progression. Newby et al. (2009) suggested that plaque growth or rupture depends on the expression of types of MMPs and the stage of plaque development. We hypothesize that pharmacological inhibitors of MMP‐9 and EGFR decrease the expression of MMPs and increase collagen content of carotid plaques. To investigate a possible link between EGFR, MMP‐1, and MMP‐9 for the loss of collagen in atherosclerotic plaques, the EGF‐treated VSMCs were grown in the presence of pharmacological inhibitor of MMP‐9 and EGFR. We demonstrate that MMP‐9 inhibitor significantly increased Col I(*α*1) and Col III(*α*1) mRNA expression and decreased EGFR, MMP‐9, and MMP‐1 mRNA transcripts in EGF‐treated VSMCs isolated from both AS and S carotid plaques compared to untreated cells. This suggests that MMP‐1 and MMP‐9 may play a significant role in the destabilization of mature collagen. A number of MMP inhibitors such as BB‐94 (batimastat) and BB‐2516 (marimastat) have been investigated in various clinical conditions without much success. It is suggested that MMP inhibitors could be beneficial in vascular dysfunction and vascular disease states in which tissue remodeling plays an important role (Raffetto and Khalil 2008). Recent studies showed that pharmacological inhibition of collagenase‐3 substantially increased plaque interstitial collagen content in mouse intima and in the fibrous cap compared to vehicle‐treated controls (Quillard et al. 2011). Selective inhibition of MMP‐12 was reported to retard atherosclerosis progression with a more fibrous plaque in mice (Johnson et al. 2011). We also demonstrate that inhibition of EGFR with AG1478 significantly decreased MMP‐1 and MMP‐9 and increased Col I(*α*1) and Col III(*α*1) mRNA expression in EGF‐treated VSMCs isolated from both S and AS carotid plaques when compared to untreated cells.

In conclusion, these results establish a mechanism by which MMP‐1 and MMP‐9 induced by EGFR activation decreases the interstitial collagens leading to plaque instability in patients with carotid stenosis and provides a direction for a translational approach to this concept. This study also provides biochemical and molecular evidence that a selective blockade of both EGFR and MMP‐9 may be a novel strategy and a promising target for stabilizing vulnerable atherosclerotic carotid plaques.

## Acknowledgments

We would like to thank Dane Marvin for his assistance with the editing of this manuscript.

## Conflict of Interest

None declared.
